# Response of imported malaria patients to antimalarial medicines in Sri Lanka following malaria elimination

**DOI:** 10.1371/journal.pone.0188613

**Published:** 2017-11-28

**Authors:** Priyani Dharmawardena, Chaturaka Rodrigo, Kamini Mendis, W. M. Kumudu T. de A. W. Gunasekera, Risintha Premaratne, Pascal Ringwald, Deepika Fernando

**Affiliations:** 1 Anti Malaria Campaign, Sri Lanka, Narahenpita, Colombo, Sri Lanka; 2 Department of Pathology, School of Medical Sciences, University of New South Wales, Sydney, NSW, Australia; 3 Department of Clinical Medicine, Faculty of Medicine, University of Colombo, Colombo, Sri Lanka; 4 Former Coordinator of the Global Malaria Program, World Health Organization, Colombo, Sri Lanka; 5 World Health Organization, Geneva, Switzerland; 6 Department of Parasitology, Faculty of Medicine, University of Colombo, Colombo, Sri Lanka; Institute of Tropical Medicine, JAPAN

## Abstract

After eliminating local malaria transmission and being certified as a malaria-free country, Sri Lanka is facing the challenge of imported malaria. At the same time, the country has the unique opportunity to be a case study for other countries in a similar situation by approaching this issue systematically, guided by evidence. This study demonstrates the importance of developing a mechanism to detect imported malaria and adopting an evidence-based approach to study the resistance of imported malaria to anti-malarial medicines. This is a prospective study of patients diagnosed with imported malaria in Sri Lanka and treated according to the national treatment guidelines, over 24 months (2015/2016). The clinical features, time to diagnosis, origin of the infection, infecting species, parasite density and the treatment given were recorded. All patients were followed up for 28 days, and in the case of *Plasmodium vivax* and *P*. *ovale* infections, the follow up period was extended to 12 months to establish treatment failures and relapses. Fifty nine uncomplicated and 15 severe imported malaria cases were reported in Sri Lanka during the study period. Most of these infections originated in either Sub-Saharan Africa or South and Southeast Asia. Having a *P*. *vivax* infection and low parasitic counts were significantly associated with relative diagnostic delay. One of the 14 uncomplicated *P*. *falciparum* patients and two of the 12 severe *P*. *falciparum* malaria patients who were followed up till day 28 had a late clinical failure. The others responded adequately to treatment both clinically and parasitologically. There was no treatment failure reported amongst any other species. This study, which is the first to assess the therapeutic response of imported malaria in Sri Lanka after elimination, demonstrates that the current antimalarial treatment policies and strategies in Sri Lanka have been effective against infections acquired overseas up until the end of year 2016.

## Introduction

Sri Lanka was declared a malaria-free country by the World Health Organization in September 2016[1]. Currently, all malaria cases reported to the country are acquired overseas (imported malaria). Due to the high receptivity and vulnerability which exists in the country, as a strategy to prevent the re-introduction of malaria, it is mandatory to confirm the diagnosis with microscopy and or RDT, and presumptive treatment is not recommended. Further, all the cases reported are investigated fully and reviewed by a panel of independent malaria experts with regard to the case management and classification[2]. All malaria patients diagnosed in the public or private health sector are treated in accordance with the national treatment guidelines issued by the Anti Malaria Campaign (AMC) of Sri Lanka[3] using quality assured antimalarial medicines.

To date, on a global scale, resistance to currently used antimalarials, including partner drugs used in artemisinin combinations has been documented in two of the five malaria species known to affect humans in nature: *Plasmodium falciparum* and *P*. *vivax*[4]. The current recommendations for the treatment of malaria in Sri Lanka may not be efficacious against all cases of imported malaria. The challenge of drug resistance in the post elimination phase cannot be ignored, and treatment guidelines need to be regularly reviewed and modified according to the resistance patterns observed with imported malaria. Therefore collecting data on antimalarial drug resistance and ascertaining the origin of infection in all cases including the drug efficacy profile in the countries of origin are important to update local treatment guidelines.

Prior to the commencement of the study, in 2013–2014, 144 imported malaria cases were reported in Sri Lanka, and 89 (62%) of these had been acquired from South and Southeast Asian countries (India, Pakistan and Myanmar). The second largest source of imported malaria to Sri Lanka was the continent of Africa. The current study on therapeutic efficacy of Sri Lanka’s first-line antimalarial medicines was deemed necessary to inform the most appropriate treatment policies for the country. The receptivity to malaria is high in Sri Lanka as the main vector *Anopheles culicifacies* is abundant in the environment favouring onward transmission from an index case[5]. Continuing malaria infections as a result of poor response to medicines could lead to onward transmission and may trigger malaria outbreaks in the population which is now largely non-immune, heralding the re-introduction of malaria to the country.

All main countries from which imported malaria cases had entered Sri Lanka (India, Pakistan and Myanmar), have endemic CQ resistant *P*.*vivax* infections[6–11]. This plus the fact that CQ is currently recommended as a suitable prophylaxis for Sri Lankans travelling to some overseas destinations, make it essential to observe the treatment failures with CQ when used for imported non-falciparum malaria cases reported in Sri Lanka.

Primaquine is being used as the hypnozoitocidal agent in Sri Lanka and the AMC recommends 0.25 mg of primaquine base/kg/day daily for 14 days for radical cure of *P*.*vivax* malaria [3, 12]. Currently there is no evidence for the adequacy of this dose for imported malaria especially as the WHO recommends a dose of 0.5 mg/kg body weight for persons acquiring malaria from Oceania and Southeast Asia[13]. Therefore this study also assessed the efficacy of primaquine in achieving radical cure for imported *P*.*vivax* and *P*.*ovale* malaria cases.

Artemisinin combination therapy (ACT) is the first line treatment for *P*. *falciparu*m malaria in Sri Lanka and in most other countries where the disease is endemic. Resistance of *P*. *falciparum* to artemisinin and its partner medicines[14] is prevalent, and originated in Southeast Asia [15, 16]. A recent report on an artemisinin "resistant" strain from Africa [17] fell short of a convincing demonstration of resistance by WHO standards [18]. Yet, it highlights the need for vigilance in the emergence of artemisinin resistance elsewhere in the world. Therefore given the current state of response to antimalarials of both *P*.*falciparum* and *P*.*vivax* globally, it is necessary that Sri Lanka monitors the therapeutic efficacy of its first line antimalarials to imported malaria for the purposes of informing drug policy and thereby preventing the re-introduction of malaria to the country.

## Methods

Ethical approval for the study was obtained from the Ethics Review Committee of the Faculty of Medicine, University of Colombo (EC 14–165) and the Ethics Review Committee of the Sri Lanka Medical Association (ERC/13-053).

This study reports cases of imported malaria from January 2015 to December 2016 (24 months) in Sri Lanka and gives a brief description of epidemiological characteristics and the response to anti malarial medicines in patients with a confirmed diagnosis of malaria during this period [3].

Parasitologically confirmed malaria patients reported during the 24- month study period were included in the study. Informed written consent was obtained from all enrolled patients (and from the parent or guardian if the subject was a minor). Every patient diagnosed with malaria was managed as per national guidelines with quality assured drugs [3]. Following treatment with the current first-line anti-malarials, the patients were followed up both clinically and parasitologically for three days (D0-D3) in a hospital as in-ward patients, and thereafter on days 7, 14, 21, and 28. If the individual failed to report, they were visited at home. The day of enrolment into the study was the day the patient was reported to the AMC headquarters. Confirmation by microscopy by the AMC and commencement of treatment occurred on the same day. *P*. *vivax* and *P*.*ovale* malaria patients were followed up for a further 12 months with monthly blood smears to monitor breakthrough relapses (due to primaquine failure).

Once a patient was confirmed as having malaria (asexual parasites) by microscopic examination at AMC headquarters or the Regional Malaria Office by Giemsa staining and examination under the light microscope, another blood smear was taken for the purpose of further scrutiny and quantification of the parasitaemia according to the protocol recommended by the World Health Organization [19]. An entire thick blood smear was examined for malaria parasites under high power. Quality control of microscopy results was ensured by cross-checking both slides by an independent parasitologist. Following initiation of treatment, two blood smears were prepared each time at 12 hour intervals until the asexual parasitaemia became zero or the patient was discharged. The smears were examined at the laboratory of the AMC headquarters by two senior public health laboratory technicians who were blinded to the study subjects.

Demographic and clinical data (including weight, blood pressure, respiratory rate, pulse rate and the axillary temperature) were collected by an interviewer filled questionnaire. Clinical data were collected on admission, before the commencement of treatment and thereafter daily during the hospital stay. Body temperature before the commencement of treatment was taken as the D0 temperature. Temperature measured at 24, 48 and 72 hours after the commencement of treatment was taken as the D1, D2 and D3 temperature respectively. Haemoglobin level was measured prior to starting therapy and repeated if deemed necessary. All *P*.*vivax* and *P*.*ovale* patients were tested for G6PD status using rapid diagnostic test kits (CareStart^TM^, Accessbio, USA).

Uncomplicated *P*. *falciparum* cases were treated with 6 doses of artemether-lumefantrine (AL) scaled by age/weight as outlined in the monogram of the product. AL was given (after a glass of milk) as a tablet of 20 mg of artemether and 120 mg of lumefantrine (Novartis, Geneva, Switzerland). If there was evidence of severe malaria, intravenous artesunate was given at a dose of 2.4 mg/kg until the person could tolerate oral treatment (subject to a minimum duration of intravenous therapy of 24 hours), at which point treatment was switched to oral AL at the above dosage and continued to completion of the full 3 days of treatment. All *P*. *falciparum* patients were treated with primaquine 0.75 mg/kg as a single dose on the 3^rd^ day at the time of discharge from hospital. If the patient first presented with uncomplicated falciparum malaria and then transitioned to severe disease, they were first treated with oral artemether-lumefantine and subsequently with intravenous artesunate.

Patients diagnosed with *P*. *vivax*, *P*. *ovale* and *P*. *malariae* infection were treated with 25mg/kg of chloroquine for a period of 3 days at the dose of 10mg/kg on days one and two and 5 mg/kg on day 3. Primaquine 0.25 mg/kg daily for 14 days was issued on the 3^rd^ day at the time of discharge from hospital to patients diagnosed with *P*.*vivax* and *P*.*ovale* after excluding G6PD deficiency. *P*.*knowlesi* infection was treated with AL [20]. Patients with a temperature above 37.5 ^0^ C were given paracetamol 1g 6 hourly. Antimalarial medicines were given under direct observation of health staff. Compliance with primaquine was monitored by AMC staff during the follow up visits. The participants were observed for 30 minutes after giving the drug dose to ensure that there was no vomiting. If vomiting occurred, the full treatment dose was repeated.

The primary outcomes measured were; a) Early treatment failure (ETF), b) Late clinical failure (LCF), c) Late parasitological failure (LPF) and d) Adequate clinical and parasitological response (ACPR) as defined according to the World Health Organization guidelines [21]. The following secondary outcomes were also assessed; a) Fever clearance rate, b) Parasite clearance rate [21]. Data were entered in to SPSS statistical package (Version 22, IBM, USA) and analysed to report descriptive statistics and significant associations with any of the previously mentioned outcomes. Statistical significance was set at 0.05 and was assessed with chi square test for dichotomous outcomes, by mean difference for continuous outcomes and time to event analysis for censored time dependent data.

Ethical clearance for the study was obtained from the ethics review committee of the Sri Lanka Medical Council and the ethics review committee of the Faculty of Medicine, University of Colombo.

## Results

**Fig 1:**
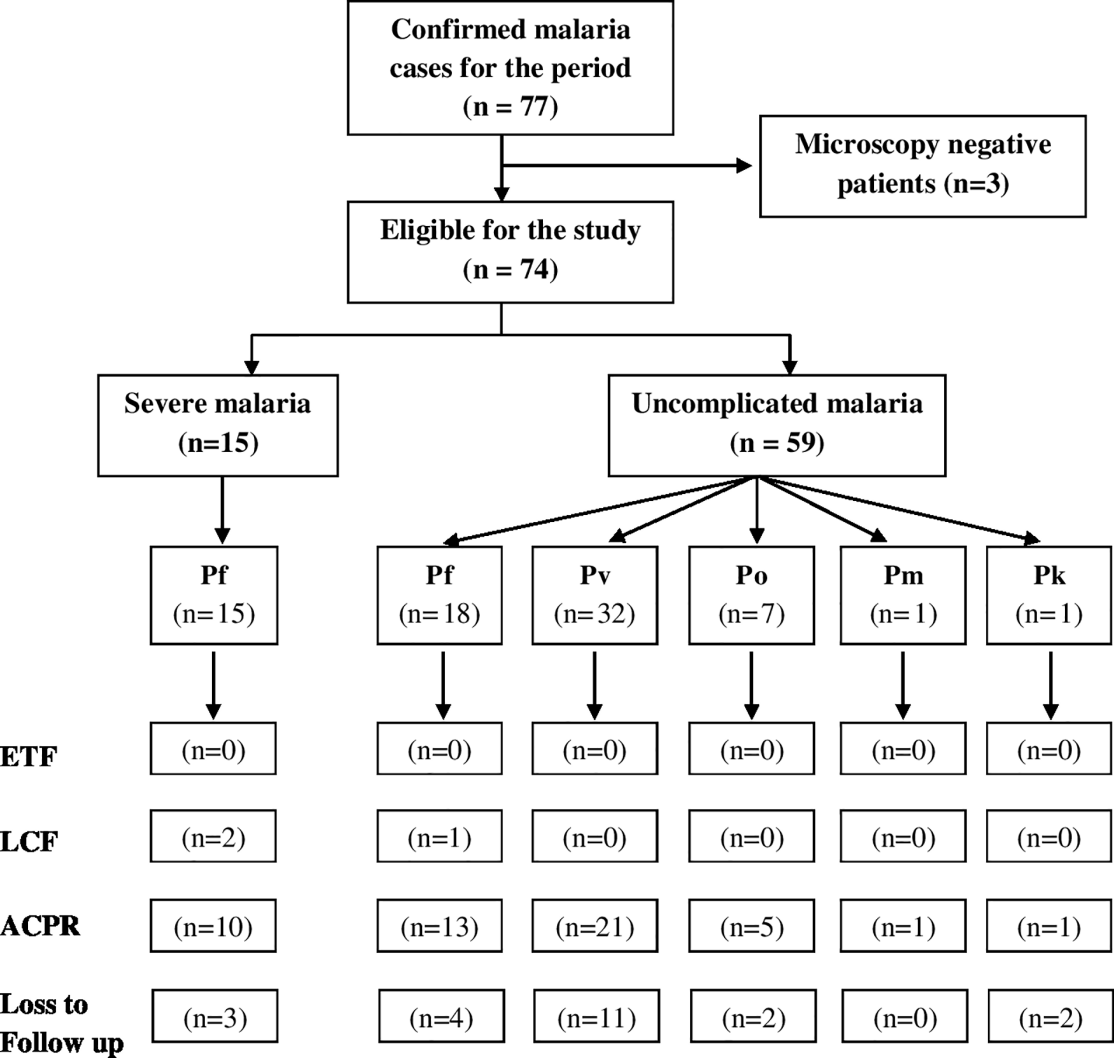
Schematic representation of subgroups of participants, attrition and outcomes.

According to the estimates of AMC, approximately 2.2 million screening tests for malaria had been performed during the study period of 24 months in Sri Lanka. These included 1,142,466 blood smears in 2015[2] and 1,072,396 in 2016 (Personal Communication, Director, AMC). A total of 77 malaria patients were confirmed by diagnosis. All malaria infections during the study period were acquired overseas as confirmed by independent experts in the Case Review Committee of AMC. Of these, three patients who were considered as cases by the expert committee were excluded from this study since their parasitaemia could not be monitored as they were positive only by rapid diagnostic testing but negative on microscopy examination (Fig 1).

**Fig 2:**
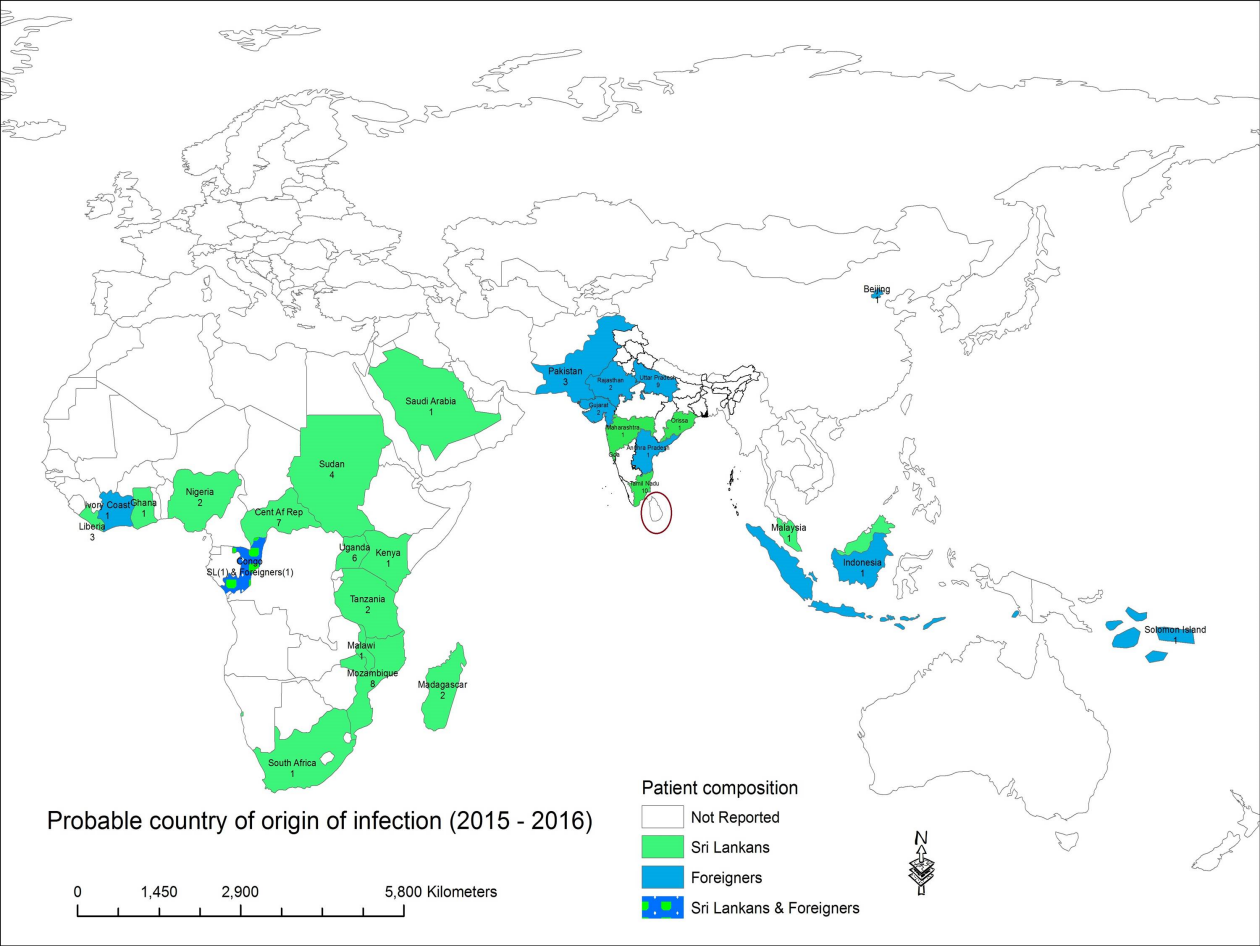
Probable country of origin of imported malaria infections in Sri Lanka (circled in red).

There were 59 patients with uncomplicated malaria and 15 patients with severe malaria included in the study. Males comprised a majority (93.2%) of the study population (Table 1). Fifty two infections were reported in Sri Lankans and 22 in foreign nationals. Amongst the uncomplicated malaria cases, infection had been acquired mainly from Asia (35/59; 61.4%) and a majority of these originated in India (28/35), followed by Pakistan (n = 3). The rest of the uncomplicated malaria cases were acquired in Sub-Saharan Africa (Fig 2).

**Table 1:** Baseline characteristics of the study population.

Variable	Uncomplicated malaria(n = 59)	Severe malaria (n = 15)
	Value (*%*) or Median (range)	
**Sex**		
Male	55 (93.2)	14 (93.3)
Female	4 (6.8)	1 (6.7)
**Age in years**	35.5 (13–66)	42.6 (27–60)
**Parasite count at diagnosis**	2283 (32–53040)	
*P*. *falciparum*	1510 (32–53,040)	138,423 (18,985–668,000)
*P*.*vivax*	2725 (60 to 49,655)	
**Nationality**		
Sri Lankan	39 (66)	13 (87)
Foreigners	20 (34)	2 (13)
**Origin of infection**		
Asia	35 (61.4)	0
Africa	24 (40.6)	15 (100)
**Plasmodium species**		
*P*.*falciparum*	18 (30.5)	15 (100)
*P*.*vivax*	32 (54.2)	
*P*.*ovale*	7 (11.9)	
*P*.*malariae*	1 (1.7)	
*P*.*knowlesi*	1 (1.7)	

Four individuals with severe malaria (26.7%) had acquired the infection from Mozambique. One to two infections each were acquired in other African countries such as Madagascar, Uganda, Tanzania, Nigeria, Ivory Coast and Sudan. Of the two foreigners who had severe malaria, one was a tourist and the other a seaman. Neither were citizens of an African country.

Species wise, *P*.*vivax* accounted for a greater proportion of uncomplicated malaria infections (n = 32), followed by *P*.*falciparum*, *P*.*ovale* infections (n = 18 and 7 respectively). There was one case each of *P*.*malariae* and *P*.*knowlesi* infections. All except one *P*. *falciparum* infections were acquired in an African country, while all but one *P*.*vivax* infections were acquired in an Asian country. Severe malaria was solely due to *P*.*falciparum* and acquired in the African Continent.

The average duration since the onset of fever to diagnosis of uncomplicated malaria was 5.1 days (range 0–20 days) for *P*.*falciparum* malaria and 10.1 days (range 1–53 days) for *P*.*vivax* malaria. The severe malaria patients who developed symptoms following their return to Sri Lanka were diagnosed within an average of 4.3 days (SD ± 6.8, range 0–27 days) since arrival in Sri Lanka. The average time taken since the onset of symptoms to diagnosis in all patients with severe malaria was 6.6 days (SD±3.6). A Kaplan Meier survival analysis showed that time to diagnosis from the onset of illness or from arrival in Sri Lanka did not differ significantly between severe and non-severe malaria cases (data not shown). In the uncomplicated malaria cases, the parasitaemia at diagnosis in *P*.*falciparum* ranged from 32–53,040/ μl (median: 1510/μl) and in *P*.*vivax*, from 160 to 49,655/µl (median: 2725/µl). In severe malaria cases the parastiaemia at diagnosis ranged from 18,985–668,000/ μl (median: 138,423.4/μl) (Table 1).

With the exception of three uncomplicated *P*.*falciparum* patients who were afebrile, the rest of the 56 patients presented with fever with or without chills and rigors. Most of the severe malaria patients had an impaired level of consciousness (Table 2). Other presentations included; renal impairment and haematuria, acute respiratory distress syndrome (ARDS) and hyperparasitaemia, defined as a parasite count greater than 2% of red cells in areas with low transmission [22].

**Table 2:** Distribution of patients among categories of severe malaria (n = 15).

Variable	Number	Percentage
Impaired consciousness	5	33.3
Pulmonary oedema	2	13.3
Hyper-parasitaemia	2	13.3
Bleeding and acute kidney injury	2	13.3
Shock	1	6.7
Other including multiple manifestations	3	20.1

All patients had normal G6PD enzyme levels and were treated according to the standard anti-malarial regimens recommended by WHO and AMC of Sri Lanka [3, 13]. Uncomplicated *P*. *falciparum* cases were treated with the full course of artemether-lumefantrine (AL) and followed up for three days. There was no loss to follow up at the end of D3 after which they received a single dose of primaquine. All these patients except four (who left the country) were followed up until D28. Apart from a single patient who had late clinical failure, all responded to a full course of AL treatment (Table 3). This patient arrived from Mozambique. His fever and asexual parasitaemia cleared with treatment by day 2. Treatment was completed and he was afebrile and microscopically negative on routine follow up until day 14. He presented again with fever on Day 19 and was positive for *P*. *falciparum*. The diagnosis was confirmed by PCR. He was treated again with a full course of AL (20 mg of artemether and 120 mg of lumefantrine) and this time responded well to treatment.

**Table 3:** Treatment outcomes for patients with uncomplicated malaria (n = 59) and severe malaria (n = 15).

Outcome	Uncomplicated malaria (%)	Severe malaria (%)
Early treatment failure	0	0
Late clinical failure*	1/14 (7.1)	2/12 (16.6)
Late parasitological failure	0	0
Adequate clinical and parasitological response	41/42 (97.6)	10/12 (83.3)

*Only observed with *P*. *falciparum* infections

With the exception of three patients diagnosed with *P*. *vivax* malaria who left the country within the first 72 hours, the rest completed their chloroquine treatment under direct observation by hospital or AMC staff. Another seven *P*.*vivax* patients left the country before completing the 14 day course of primaquine. Two *P*.*ovale* patients were lost to follow up by day 14. All other patients with *P*. *vivax* (n = 22) and *P*. *ovale* (n = 5) infections completed their 14 day primaquine course. One other *P*.*vivax* patient was lost to follow up by D28. The treatment efficacy at D28 for the non-falciparum infections was 100%. Eleven (11/21, 50%) *P*.*vivax* malaria patients and four (4/5, 80%) *P*.*ovale* patients completed the 12 month follow up, and none developed a relapse. Two *P*.*vivax* patients are still being followed up and the remainder (8/21, 38.1%) were lost to follow up as they left the country.

Regarding the secondary outcomes in the 56 patients with uncomplicated malaria, who were febrile at presentation, the fever clearance rate was 92.8% (52/56) by D3. All cases were afebrile by D4. The parasite clearance rate was 94.9% (56/59) by D3. Except for the *P*.*malariae* patient who had asexual parasitaemia till D7, other two patients who were positive for *P*.*falciparum* showed zero parasitaemia on D4.

**Fig 3:**
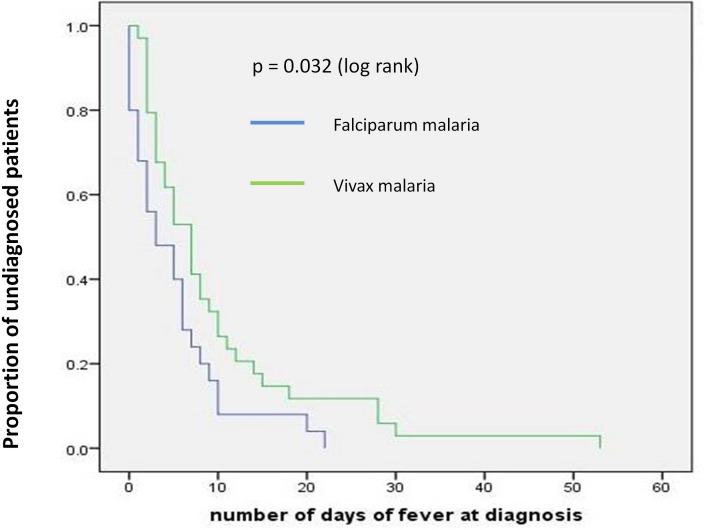
Time to diagnosis categorized according *Plasmodium* species.

**Fig 4:**
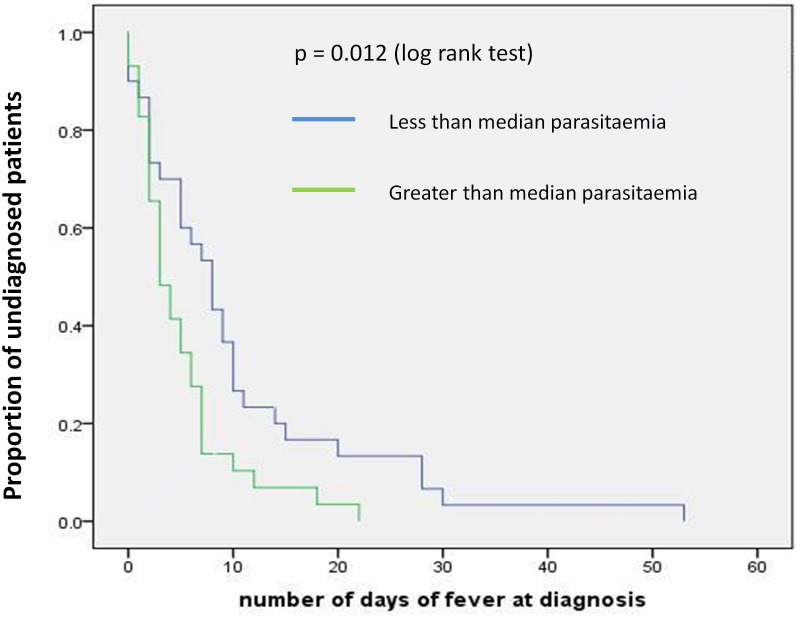
Time from onset of symptoms to diagnosis according to parasite load at diagnosis.

In uncomplicated malaria patients, a Kaplan Meier survival analysis showed that there was no significant difference in parasite clearance time or fever clearance time when subjects were grouped according to the infecting *Plasmodium* species or the initial parasite count (less or more than median value). However time to diagnosis (since the onset of symptoms) was significantly longer when the patients had *P*.*vivax* malaria (as opposed to *P*. *falciparum* malaria) (Fig 3) or when the patient had a parasitic count less than the median value (p<0.05, Fig 4).

For patients with severe malaria, treatment was commenced with IV artesunate in seven individuals and in the other eight, treatment was initiated with oral artesunate but soon replaced with IV artesunate. Early treatment failure was not observed in any of the patients with severe malaria. A majority (12/15, 80%) had a fever and parasite clearance time of less than 72 hours and all were both clinically and parasitologically cleared by D7. Two of the patients (both Sri Lankans) had late clinical failure with a recurrence of symptoms and parasitaemia at D15 and D19 respectively after an initial “apparent” cure. The presumed source of infection was Congo and Madagascar respectively. In both these patients the initial infection was treated with 3 doses of IV artesunate at a dose of 2.4 mg/kg at which point they were able to tolerate oral medication and thereafter a full 3 day course of AL was administered. The recurrent infections in both patients were re-treated with another three doses of intravenous artesunate followed by a full course of oral AL and both patients responded to treatment clinically and parasitologically. The two foreign patients with severe malaria left the country between D3-D14 after attaining early treatment success so their outcome with regard to late clinical failure is unknown, as with one Sri Lankan. Thus three patients with severe malaria were lost to follow up by day 28. Overall, adequate parasitological and clinical response to this case series was (10/12; 83.3%) by D28 (Table 3). There was no significant difference in this figure when compared to patients with uncomplicated *P*.*falciparum* malaria.

## Discussion

This study assessed the clinical and parasitological response to recommended treatment schedules of imported malaria in Sri Lanka over a 24 month period. Due to the low number of malaria cases being reported in Sri Lanka (and all of them being imported cases), every patient diagnosed with uncomplicated malaria by microscopy (with asexual parasites) was included in this study irrespective of the parasite count at diagnosis. All attempts were made to follow up patients who remained in Sri Lanka until 28 days post-treatment (for *P*.*falciparum*, *P*.*malariae* and *P*.*knowlesi* infections) or one year post treatment (for *P*.*vivax* and *P*.*ovale* infections).

A high proportion, 15 out of 33 (45%) of patients with *P*.*falciparum* infections in this sample were classified as severe malaria, possibly an overestimate because the classification was done mainly on the basis of clinical features, in the interest of caution. The incidence of severe malaria among imported *P*.*falciparum* infections tends to be generally higher than in endemic situations [23, 24], this being likely due to the fact that travelers are most often non-immune adults, and also possibly due to delays in diagnosis. In this study however, the time from onset of symptoms to diagnosis was not significantly different between those with uncomplicated and severe *P*.*falciparum* malaria.

The response rate to treatment in uncomplicated malaria patients was excellent with only one late clinical failure being reported out of fifty nine patients. The clinical and parasitological response rates were similar in the group of severe malaria as well (only two out of 12 cases of late clinical failure). Delays in diagnosis as measured by the time from onset of illness to diagnosis was a concern, more so for *P*. *vivax* malaria patients in one of whom the diagnostic delay was nearly two months. Diagnostic delay with *P*.*vivax* malaria had been observed to be significant (compared to *P*. *falciparum* malaria) in a previous study in Thailand and this was attributed to the less severe symptomatology of the infection[25]. The faster diagnosis of an infection of *P*. *falciparum* may also be explained by a) almost all of them coming from African destinations and b) a significant proportion of them being defense force members on official duty who receive appropriate health education and systematic screening for parasitaemia upon return. It is also possible that people returning from African destinations are more conscious of the risk of malaria due to high transmission intensities in Sub-Saharan Africa and promptly seek medical advice in event of suggestive symptoms. Delay in diagnosis was also associated with lower than average parasitaemia, this may have been due to the resulting lower intensity of symptoms or even atypical symptoms which would not therefore raise a clinical suspicion of malaria. Low parasite densities may have also given rise to false negative RDT or microscopy readings in the early days of the illness leading to a delay in diagnosis.

All uncomplicated *P*. *falciparum* infections (mostly acquired in Africa) and *P*. *vivax* infections (mostly acquired in South and Southeast Asia) were highly responsive to the first line chemotherapeutic agents namely; artemether + lumefantrine or choloroquine + primaquine respectively. The only late clinical failure was reported for a *P*.*falciparum* infection acquired from Mozambique. The response to AL for *P*.*falciparum* infections in Mozambique as measured from sentinel site data is close to 100% however a few cases of artemisinin resistance have been reported [26]. As illustrated by this study, monitoring of therapeutic efficacy in travellers in countries with no endemic malaria can be quite challenging due to the fact that they are people on the move, resulting in a high attrition rate. However due to the rarity of the disease, it is also logistically easier to follow up smaller numbers. Given the fact that residents of malaria free countries such as Sri Lanka may have no acquired immunity against malaria, such patients can serve as early indicators of emerging resistance to currently prescribed anti-malarial regimens when they present as imported malaria cases.

Sri Lanka was declared malaria free in September 2016 but all of its South Asian neighbours except the Republic of Maldives[27] are endemic for malaria at present. The number of tourists visiting Sri Lanka has tripled during the period from year 2000 to 2013 from 400,000 per annum to 1,300,000 per annum[28]. This does not include foreigners who visit for business, study or work. Similarly the number of Sri Lankans travelling overseas for purposes of leisure, business, study and work has been increasing. Estimates at the last national census in 2011 revealed that over 600,000 Sri Lankans live abroad for 6 months or longer [29]. The number of people going overseas and returning for short visits is expected to be much higher. A large proportion of these travelers departs to and returns from India, which has endemic malaria transmission. In year 2013, approximately 500,000 individuals crossed the borders of these two countries and it is a 37.5% increase compared to 2008[30]. Comparatively, a much smaller exchange occurs between African countries and Sri Lanka. However, a large proportion of malaria cases detected in this study originated from Africa which shows that this route is highly vulnerable to the acquisition of malaria. Since 2009, Sri Lanka has been sending its defense force members to war-torn African nations such as the Central African Republic and Liberia for United Nations peacekeeping missions[31]. A close liaison between the AMC and the Ministry of Defense ensures that all soldiers posted in overseas endemic territories receive appropriate chemoprophylaxis and health advice. In addition, all returnees are screened for malaria at the ports of entry to Sri Lanka[32]. Despite these preventive measures, there have been outbreaks of malaria (including fatalities) among Sri Lankan defense force members while serving overseas[32]. Also, a number of returnees were diagnosed with malaria when screened at the airport upon return. These measures have been in place even before Sri Lanka eliminated malaria, and the smooth transition to a malaria free Sri Lanka highlights the success of these preventive strategies. However, currently there is no such screening for civilian travelers (or defense force / police personnel on personal overseas travel). In these situations screening is voluntary. AMC provides free travel advice and prophylactic medicines for up to six months for any member of the public travelling to malaria endemic countries, and also maintains free screening services at all air and sea ports. The success of these strategies depends on appropriate health education of the public which is a challenge in a low incidence environment where malaria is no longer considered as a “cause of concern”. Previously we have demonstrated several instances where diagnostic delays have occurred due to misdiagnosis, lack of awareness and non-suspicion of malaria as a differential diagnosis by treating physicians[33, 34]. This requires raising awareness and preparedness for diagnosis on the part of the treating physicians as well as the general public.

Our results indicate that current anti malarial treatment regimens offered in Sri Lanka are effective against all species of malaria among travellers to the country at present. The only treatment failure observed in uncomplicated disease was in a Sri Lankan returning from Mozambique infected with *P*. *falciparum*. These observations suggest that routine follow up of patients with *P*. *falciparum* malaria is essential at least until 28 days since beginning of treatment. Regarding *P*.*vivax* malaria, there were no relapses reported in any of the treated subjects suggesting the adequacy and efficiency of the current radical cure strategies. Both *P*. *ovale* and *P*. *malariae* infections identified during this study responded to the medicines used. Neither species were endemic in Sri Lanka prior to malaria elimination.

### Limitations

The attrition rate during follow up was high for several subgroups. This was especially evident in the case of *P*.*vivax* malaria where only 11 patients completed their 12 month follow up. Hence the efficacy of primaquine in attaining radical cure cannot be established with certainty. It was not possible to improve these numbers as all foreigners and most of the locals, who defaulted, had left Sri Lanka.

## Conclusions

After cessation of local transmission of malaria, Sri Lanka faces the challenge of tackling imported malaria. Since the receptivity to malaria is high in the country with the vector *Anopheles sp*. found in abundance, it is important to diagnose early and effectively cure all imported malaria cases to prevent re-establishment of local transmission. This has to be guided by regular monitoring of the species profile of infections, the country of their origin, the time taken to diagnosis, response to medicines and relapse patterns during follow up. The current study which examined these parameters in patients during 2015 and 2016, suggests that a) the prevailing treatment schedules and strategies have been effective against infections imported during the study period; b) there are unacceptable delays in diagnosis of malaria particularly in the case of vivax malaria, and c) *P*. *falciparum* infections, almost all of which originated in sub-Saharan Africa, need to be followed up (at least up to 28 days since initiation of treatment) to detect late clinical / parasitological failures. International non-immune travelers acquiring malaria overseas is a useful group to monitor treatment efficacy to guide treatment policies after elimination of malaria. They also serve as a sensitive sampling pool to detect changes in parasite response to medicines in endemic countries where they got infected from.

## Abbreviations

ACPR: Adequate clinical and parasitological response
ACT: Artemisinin, combination therapy
AL: Artemether-lumefantrine
AMC (HQ): Anti Malaria Campaign (headquarters)
CQ: Chloroquine
ETF: Early treatment failure
FCT: Fever clearance time
IV: Intravenous
LCF: Late clinical failure
LPF: Late parasitological failure
PCR: Polymerase chain reaction
PCT: Parasite clearance time
RDT: Rapid diagnostic testing
WHO: World health organization
